# Genome-wide tracts of homozygosity and exome analyses reveal repetitive elements with Barrets esophagus/esophageal adenocarcinoma risk

**DOI:** 10.1186/s12859-019-2622-y

**Published:** 2019-03-14

**Authors:** Visanu Wanchai, Jing Jin, Emine Bircan, Charis Eng, Mohammed Orloff

**Affiliations:** 1 Arkansas Center for Genomic Epidemiology & Medicine and The Department of Biomedical Informatics, University of Arkansas for Medical Sciences, Little Rock, AR 72205 USA; 2 The Department of Epidemiology, University of Arkansas for Medical Sciences, Little Rock, AR 72205 USA; 3 Winthrop P. Rockefeller Cancer Institute, University of Arkansas for Medical Sciences, Little Rock, AR 72205 USA; 4 Genomic Medicine Institute, Cleveland Clinic, Cleveland, OH 44195 USA

**Keywords:** Barrett’s esophagus, Esophageal adenocarcinoma, Tracts of homozygosity, Exome, Omics

## Abstract

**Background:**

Barrett’s esophagus (BE) is most commonly seen as the condition in which the normal squamous epithelium lining of the esophagus is replaced by goblet cells. Many studies show that BE is a predisposing factor for the development of esophageal adenocarcinoma (EAC), a particularly lethal cancer. The use of single nucleotide polymorphisms (SNPs) to map BE/EAC genes has previously provided insufficient genetic information to fully characterize the heterogeneous nature of the disease. We therefore hypothesize that rigorous interrogation of other types of genomic changes, e.g. tracts of homozygosity (TOH), repetitive elements, and insertion/deletions, may provide a comprehensive understanding of the development of BE/EAC.

**Results:**

First, we used a case-control framework to identify TOHs by using SNPs and tested for association with BE/EAC. Second, we used a case only approach on a validation series of eight samples subjected to exome sequencing to identify repeat elements and insertion/deletions. Third, insertion/deletions and repeat elements identified in the exomes were then mapped onto genes in the significant TOH regions. Overall, 24 TOH regions were significantly differentially represented among cases, as compared to controls (adjusted-*P* = 0.002–0.039). Interestingly, four BE/EAC-associated genes within the TOH regions consistently showed insertions and deletions that overlapped across eight exomes. Predictive functional analysis identified NOTCH, WNT, and G-protein inflammation pathways that affect BE and EAC.

**Conclusions:**

The integration of common TOHs (cTOHs) with repetitive elements, insertions, and deletions within exomes can help functionally prioritize factors contributing to low to moderate penetrance predisposition to BE/EAC.

**Electronic supplementary material:**

The online version of this article (10.1186/s12859-019-2622-y) contains supplementary material, which is available to authorized users.

## Background

Barrett’s esophagus, the only known precancerous lesions for esophageal adenocarcinoma (EAC), is characterized as an abnormal replacement of the stratified squamous epithelium in the lower portion of the esophagus with metaplastic, columnar epithelium called goblet cells that can secrete gel-forming mucins [1]. Esophageal cancer is more common among men than women [2]. According to the American Cancer Society’s estimate for 2018 [2], the lifetime risk of developing esophageal cancer in the United States is about 1 in 132 for men and about 1 in 455 for women.

Of the two cancer types, EAC and squamous cell carcinoma (SCC), EAC is more prevalent in the United States overall [2], while SCC is observed more often in African Americans [3]. Possible risk factors, other than race, have been identified by previous studies; these include aging, male gender, gastroesophageal reflux disease, tobacco use, alcohol use, diet, and human papillomavirus (HPV) [2, 4].

Somatic mutations in EAC have been studied and some of the common mutations have been identified in *TP53*, *CDKN2Ai*, *BRAF*, *CTNNB1*, *EGFR*, *KRAS*, *PTEN* and *CDH1* [2]. Although most cases of BE and EAC are believed to be sporadic, heritable etiologies have been reported as well [5, 6]. In addition to shared environmental factors, reports on BE and EAC have shown a frequent autosomal dominant mode of inheritance with incomplete penetrance and autosomal recessive inheritance, which are rare [7].

Historically, several types of genetic markers, including SNPs, have been used to map genes to BE/EAC [8]. Polymorphic tracts of nucleotide sequences, such as those of repeat nucleotides of variable lengths [9, 10] and tracts of homozygosity (TOHs) [11–15], seem to occur frequently in the human genome. Whereas the importance of SNPs and their associations with disease risk are well established [16, 17], there is an increasing appreciation for the potential role of nucleotide repeats [9, 18] and TOHs [11, 14, 15, 19, 20] in the risk of developing disease. Nucleotide repeats and TOHs are common in the genome; however, their associations with risk of developing common diseases are understudied [21].

Approximately half of the human genome is composed of highly repeated DNA sequences [22]. Some of these nucleotide repeats (e.g., satellite repeats) have been shown to transcribe into noncoding RNAs, which have been linked to gene silencing and maintenance of chromosomal integrity [23]. The processes that involve inversions and deletions in the genome yield another family of repeats called inverted repeats (or IRs), which are a single stranded sequence of nucleotides that are followed downstream by their reverse complement [24]. If selection pressure favors minimizing inversions, then more direct repeats are expected, relative to IRs [23, 25–28]. However, inversions are required to create IRs from direct repeats, if these repeats originate mainly from close direct repeats [29]. The sequences of these IRs have been found to locate near endogenous chromosomal instability and breakage hotspots [21], but the mutagenic potential of IRs has not been well characterized. To the best of our knowledge, the role of repeat sequences in BE/EAC is understudied.

Several genomic studies have investigated the genetic susceptibility of BE/EAC [5, 30–33]. Whole-exome sequencing studies have investigated the genomic alterations in a larger sample size and reported mutations in *ELMO1* and *DOCK2* [5, 34]. BE/EAC has persistently displayed heterogeneous clinical outcomes with an underlying genetic heterogeneity. In the study reported here, we attempt to merge multiple types of genomic changes, including repeats, TOHs, and insertions/deletions, across platforms to better understand the progression of BE to EAC.

## Results

### Prediction of common TOH regions

The common TOH (cTOH) regions were identified as described by Orloff et al. [35]. After false-discovery rate (FDR) adjustment for the effects of sex and population stratification factors, 24 cTOH regions on 13 chromosomes were found to be significantly differentially represented between BE/EAC cases and controls with *P* < 0.05 (Table 1). There are 13 cTOH regions that are over-represented in the BE/EAC cases, as compared to controls, with odds ratios (OR) 2.38–8.36 (adjusted-*P* = 0.002–0.045). In addition, there are 11 cTOH regions under-represented in BE/EAC cases, as compared to controls (OR = 0.15–0.48, adjusted-*P* = 0.004–0.038). The largest region of cTOH is on chromosome 13 that covers four Mbp and harbors the most number of SNPs (Table 1). The smallest cTOH region was identified on chromosome 20, the smallest chromosome.

**Table 1:** All predicted cTOH regions

Chromosome regions	Length (bp)	No. of SNPs	Adjusted *P*-value	Adjusted FDR assoc.	Adjusted OR (95%CI)
1p22.3	381,139	113	0.025	0.653	4.65 (1.22,17.81)
2p23.3 - p23.2	1,363,418	165	0.021	0.888	0.32 (0.12,0.84)
3p24.3	1,039,742	238	0.015	0.315	2.78 (1.22,6.32)
3p24.3	434,675	122	0.01	0.315	8.36 (1.66,42.09)
3p24.1	628,410	100	0.023	0.315	0.21 (0.06,0.81)
3q13.32	1,263,994	227	0.028	0.315	2.67 (1.11,6.41)
4q12	434,403	115	0.034	0.625	3.89 (1.11,13.66)
4q13.1	879,986	150	0.026	0.625	0.48 (0.25,0.91)
6p12.3	1,416,850	181	0.03	0.554	0.39 (0.17,0.92)
6q14.1	384,341	102	0.039	0.554	5.3 (1.09,25.82)
7p22.2	632,597	187	0.045	0.707	2.84 (1.03,7.85)
7p12.2	462,813	129	0.031	0.707	0.34 (0.13,0.9)
8q21.11 -q21.12	1,727,694	238	0.023	0.653	2.72 (1.15,6.43)
8q22.2	1,589,641	117	0.029	0.653	0.25 (0.07,0.87)
9q21.13	1,308,361	255	0.036	0.333	0.46 (0.22,0.95)
9q21.31	491,323	107	0.026	0.333	0.25 (0.08,0.85)
9q33.1	958,487	265	0.013	0.333	2.48 (1.21,5.08)
9q34.13	367,627	107	0.037	0.333	0.18 (0.04,0.9)
12q14.1	1,242,069	168	0.038	0.611	0.37 (0.15,0.95)
13q21.1	4,413,655	608	0.002	0.05	2.38 (1.36,4.16)
14q13.3-q21.1	866,302	149	0.012	0.252	3.1 (1.28,7.49)
15q22.33-q23	1,142,927	150	0.023	0.368	4.15 (1.22,14.12)
18q12.1	584,602	124	0.004	0.052	0.15 (0.04,0.54)
20p12.1	267,272	104	0.029	0.29	4.22 (1.16,15.39)

### Distribution of IRs in the cTOH regions

The significant 24 cTOH regions were used as a guide to screen for the presence of repeats, and more specifically IRs, using exome sequence data generated from eight BE/EAC patients that served as a validation series. The search yielded 61,858 predicted IRs with an average size of 4606 bp (ranging from 39 to 21,947 bp). The most abundant of the predicted IRs were in the cTOH regions, located on chromosomes two and nine, while the least abundant IRs were located in the cTOH regions on chromosome 13 (Table 2). We found that the predicted IRs were disparately distributed within significant cTOH regions.

**Table 2:** Distribution of IRs and significant genes across cTOH regions

Chromosome	No. of IRs	Min (bp)	Max (bp)	Average (bp)	No. of genes with IRs	Significant genes^a^
chr1	1362	39	14,668	4731	10	*MCOLN2,* ***WDR63*** ^***b***^
chr2	11,340	41	11,425	5025	19	
chr3	5591	39	15,570	4512	18	*EOMES,* ***KAT2B*** ^***b***^ *,* ***RBMS3*** ^***b***^
chr4	6635	39	18,320	4451	12	*TMEM165*
chr6	4166	39	17,214	4220	11	
chr7	4219	43	11,301	4728	24	*AMZ1,* ***GNA12*** ^***b***^ *, IQCE, SDK1, SNX8, TTYH3*
chr8	4440	39	21,947	4492	12	*C8orf84, HNF4G,* ***VPS13B*** ^***b***^
chr9	10,387	39	18,110	4711	33	*C9orf98, LAMC3, NUP214, RFK, RPSAP9, TMEM2,* ***TLE1*** ^***b***^
chr12	1754	41	20,219	4064	6	*METTL1,* ***MON2*** ^***b***^ *, USP15*
chr13	1008	39	13,255	3672	0	
chr14	2186	39	19,438	4482	9	***CTAGE5*** ^***b***^ *, SEC23A, SLC25A21*
chr15	3852	39	11,243	4718	14	*AAGAB, IQCH, LRRC49*
chr18	2711	39	13,381	4319	4	*B4GALT6*
chr20	2207	39	12,552	4448	8	*C20orf7*

^a^Present in at least three out of eight samples

^b^Of the 33 genes, the pathway analysis prioritized eight genes to the NOTCH, transcription, inflammation, and signaling pathways of BE/EAC

### Distribution of insertion/deletion in cTOH regions and across all samples

**Fig. 1:**
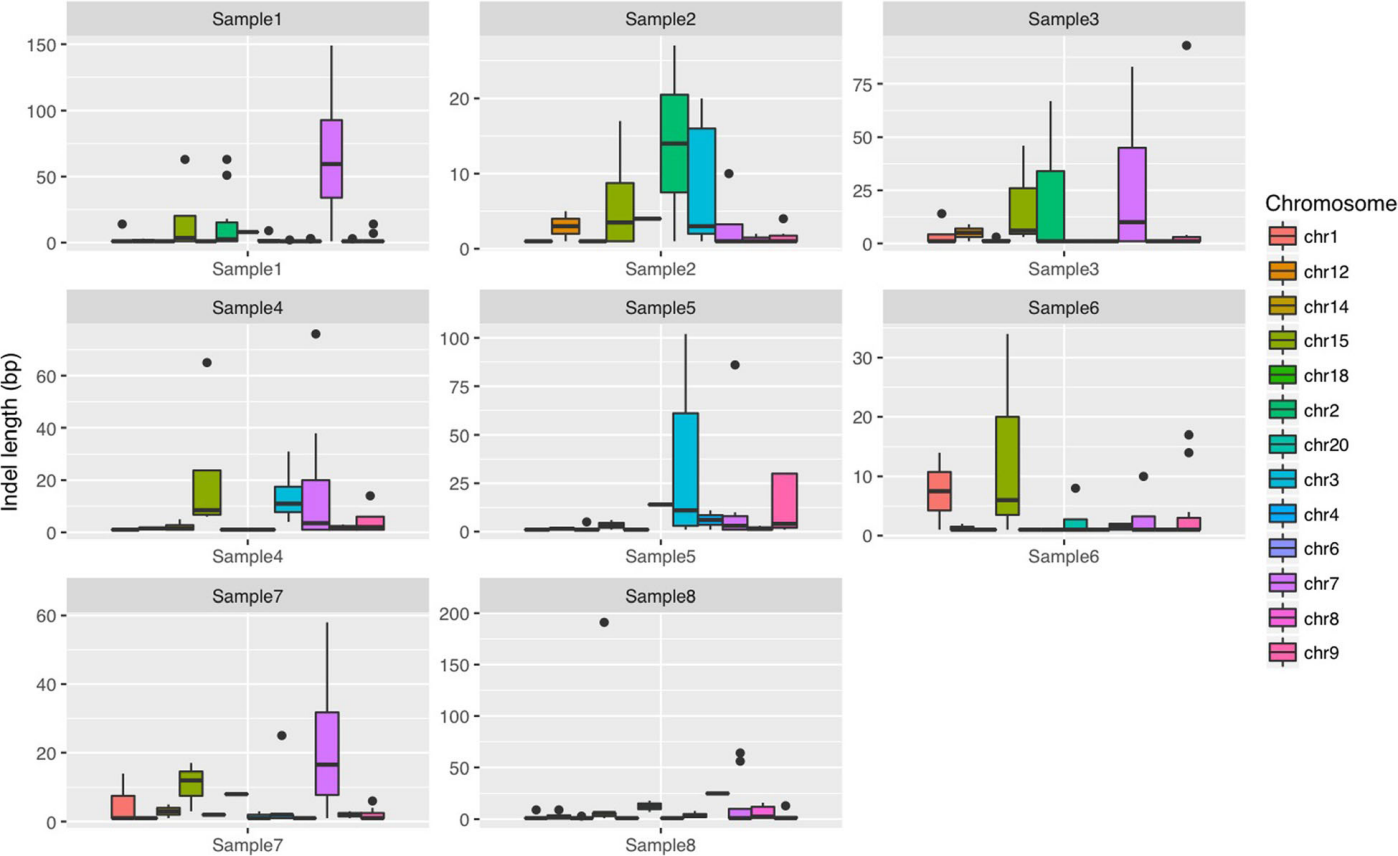
Length distribution of insertions and deletions in all samples. A display of identified insertions and deletions (or indels) within significant cTOH regions in the eight BE/EAC patients. The y-axis is the length of the nucleotide in base pairs (bp). The length of the insertions and deletions for all samples ranged from 1 to 191 bp. On the far right, a key shows the different chromosomes that harbored cTOHs, insertions, and deletions of interest

The TOH regions harbor other types of genomic variants in addition to SNPs and repeat elements. Therefore, we sought to identify insertions and deletions by using the exome sequence data generated from the eight BE/EAC patients. We identified insertions and deletions within the cTOH regions, and their distributions varied in the eight BE/EAC patients (Fig. 1). We located 180 positions of insertions and deletions on genes across all cTOH regions. The lengths of the insertions and deletions for all samples ranged from 1 to 191 bp. One of the eight samples had a very high frequency of insertions and deletions. Overall, chromosomes 7 and 15 seemed to have longer insertions and deletions in 50% of the samples. However, in one of the samples, the longest insertions and deletions were on chromosome 15 (Fig. 1). Chromosome 3 also had relatively long insertions and deletions in 38% of the samples. Shorter insertions and deletions were more frequent than longer insertions (Table 2).

### Mapping genes in significant cTOH regions that align with identified insertions/deletions and repeats using exome sequences

**Fig. 2:**
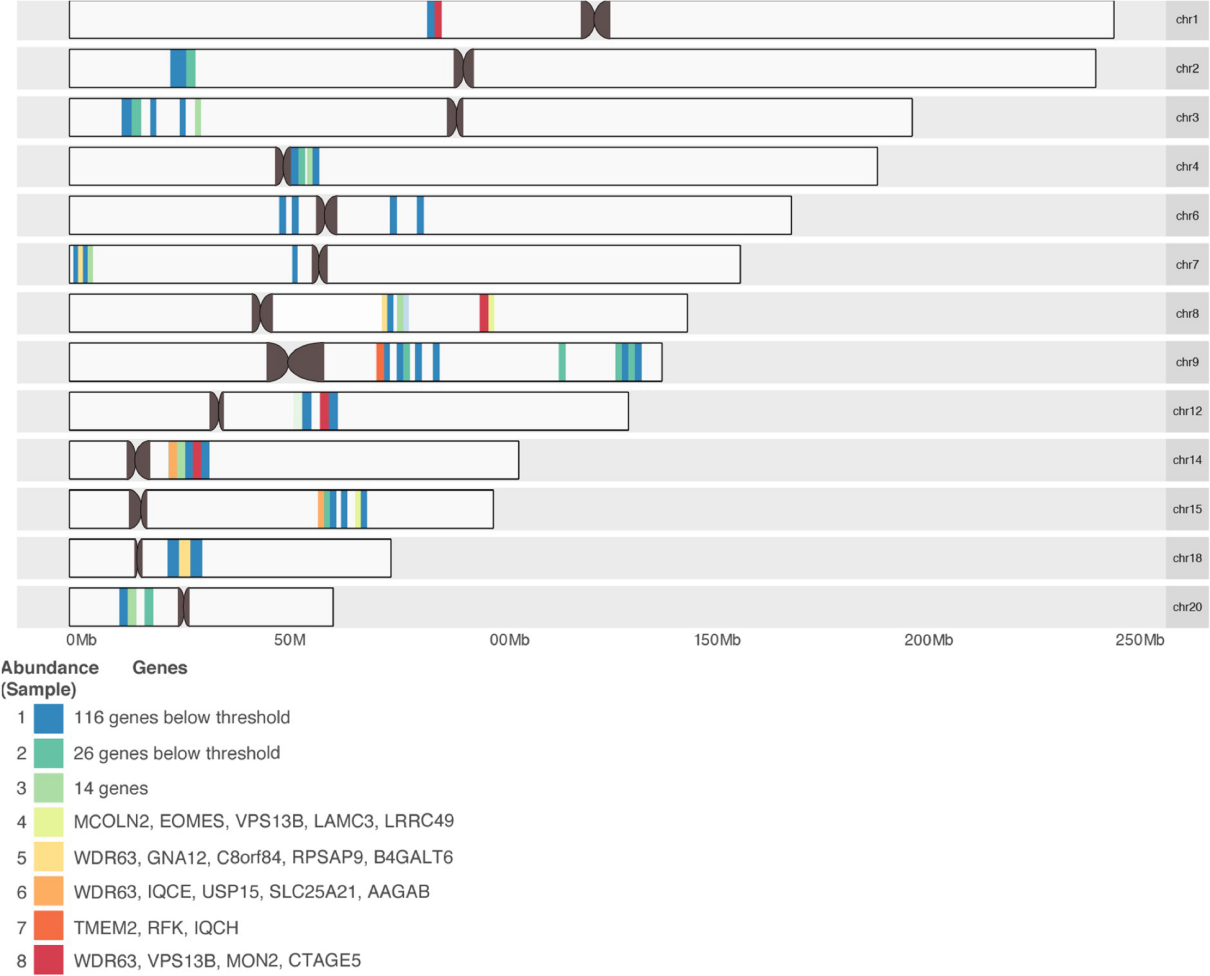
Karyogram showing mapped samples and significant genes. The approximate location of all genes within the identified cTOH regions on different chromosomes. In addition, 33 genes were prioritized, and samples four through eight harbored insertions and deletions

The identified insertions and deletions were mapped on to the genomic regions containing the 33 genes, as displayed on the karyogram in Fig. 2. The genes that consistently showed insertions and deletions that overlapped across all samples were *WDR63* (a WD repeat in domain 63)*, VPS13B* (a vacuolar protein sorting homolog 13 B)*, MON2* (a regulator to endosome-to-Golgi trafficking), and *CTAGE5* (a cutaneous T-cell lymphoma-associated antigen 1), Additional file 1. Interestingly, we identified miR-4423, located around 600 base pairs downstream of *WDR63*, which has previously been associated with airway epithelial cell differentiation and other cancers, e.g., lung cancer [36]*.*

### Further characterization of the short-listed genes and their roles in BE/EAC

**Fig. 3:**
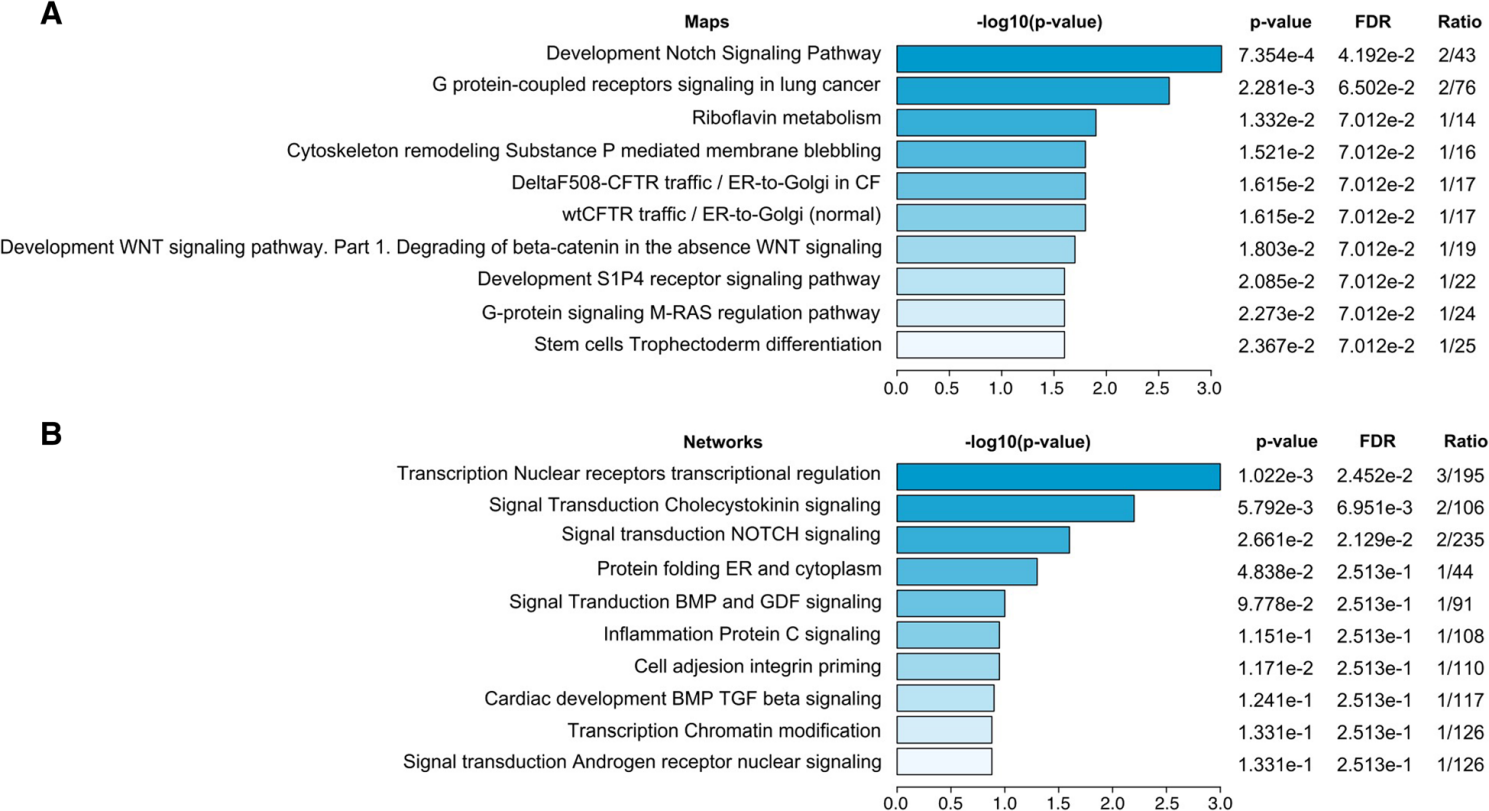
Display of pathways related to significant genes in cTOH regions. **a** Enriched pathways identified from EAC specific genes. Analysis of the key prioritized genes revealed the top ten enriched pathways. The intensity of the blue color bars reflects the importance of the pathway in BE/EAC. The top two pathways are the NOTCH signaling pathway and G protein-coupled signaling pathway. Other important pathways include the ER-to-Golgi and WNT pathways. **b** Enriched networks identified from EAC specific genes. Analysis of the key genes revealed the top ten functionally enriched process networks. The intensity of the blue color bars reflects the importance of the network processes in BE/EAC. The top nine network processes are likely important processes in BE/EAC. Transcriptional regulation and cholecystokinin signaling are the two top network processes for BE/EAC. NOTCH signaling, ER, and inflammation protein C signaling processes seem to be important in BE/EAC

The genes identified within the BE/EAC-related cTOH regions, including those that overlapped with the insertion/deletion and IRs, may have roles in either the development or progression of BE to EAC. MetaCore™ bioinformatics software was used to analyze biological pathways as well as disease and gene networks that are associated with BE/EAC. Analysis of the short-listed genes revealed the top ten enriched pathways and networks (Figs. 3a and b). The top two pathways are the NOTCH signaling pathway and G (or guanine nucleotide-binding) protein-coupled signaling (Fig. 3a), followed by the endoplasmic reticulum (ER)-to-Golgi and WNT pathways (Fig. 3A). Transcriptional regulation and cholecystokinin signaling are the two top networks identified for BE/EAC (Fig. 3b), followed by NOTCH signaling, ER, and inflammation protein C signaling, which are also important in BE/EAC (Fig. 3b).

**Fig. 4:**
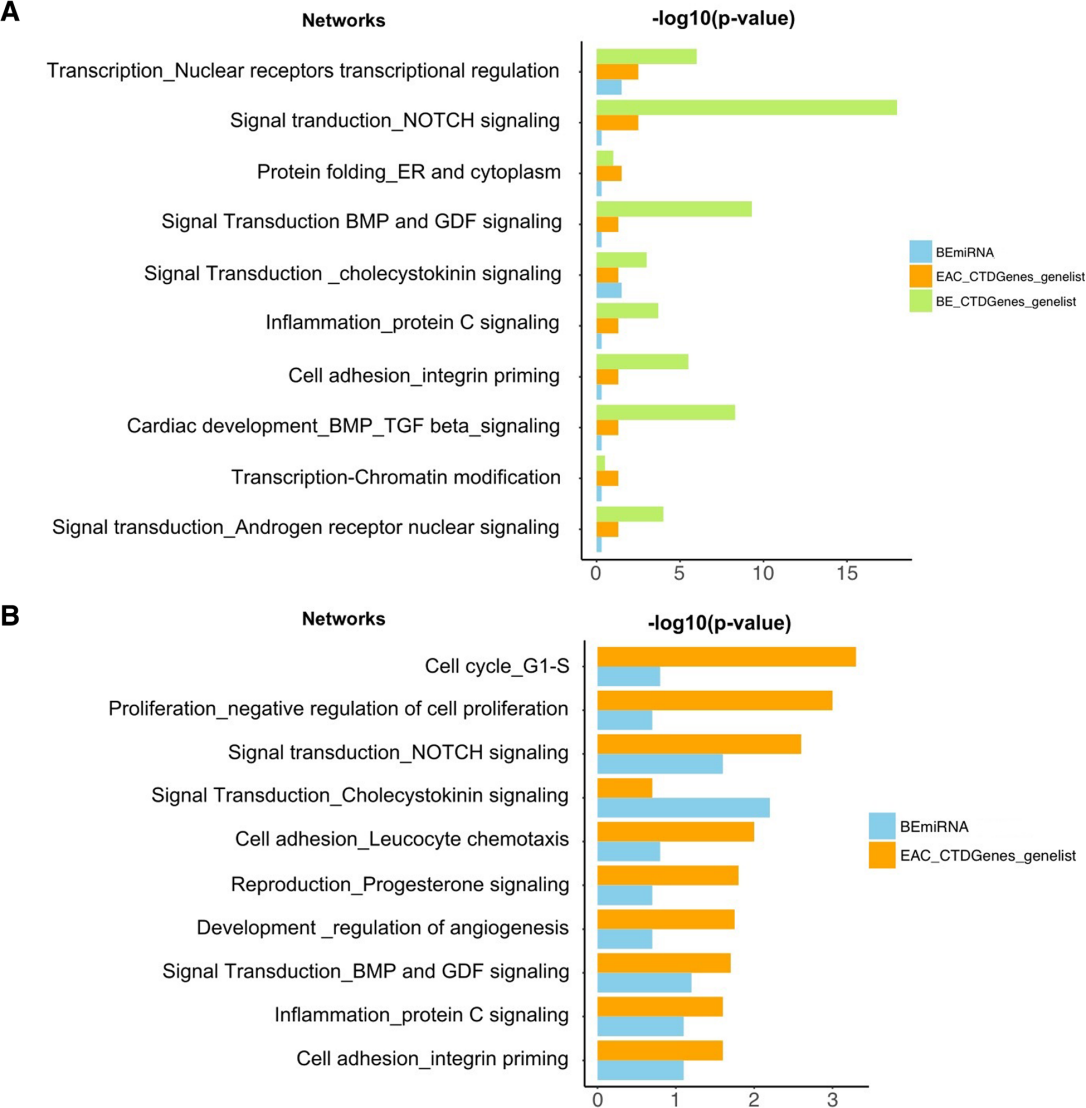
Determining relationships between BE/EAC genes and miR-4423. **a** Networks for the genes and miR-4423 in BE and EAC. Analysis of key genes using Comparative Toxicogenomics Database showed pathways similar to those identified by the MetaCore database. These pathways are affected in BE and EAC. Increased representation of a miR-4423-related (i.e., BEmiRNA in the figure) pathway inversely correlates with BE-specific pathways. Note that miR-4423 is the microRNA adjacent to *WDR63*. Overlap exists across BE, EAC, and miR-4423 pathways or processes. **b** Networks for EAC-specific genes and miR-4423. Analysis of the key genes and Comparative Toxicogenomics Database showed pathways similar to those identified by the MetaCore database that are affected by BE and EAC. Interestingly, the miR-4423-related pathway (BEmiRNA) is significantly overrepresented in cholecystokinin signaling. In general, the miR-4423 pathway is underrepresented compared to other pathways. Overlap exists across EAC and the miR-4423 pathways or processes

The use of multiple data sources can help provide comprehensive information about the functional roles of the identified genes. Therefore, in addition to MetaCore, we also used the Comparative Toxicogenomics databases to analyze all the genes and miR-4423, which yielded both complementary and supplementary results on key functional roles that affect BE and EAC, as shown in Figs. 4a and b. Some of the overlapping pathways were NOTCH, WNT, and G-protein signaling pathways. The analysis also revealed differentially affected pathways in BE and EAC.

As with the MetaCore database, NOTCH and inflammation were persistently important. Differentially affected pathways were associated with the identified miR-4423 and were overrepresented in transcriptional regulation, NOTCH and cholecystokinin signaling, cell cycle regulation, and others (Figs. 4a and b). Overlap existed across BE, EAC, and miR-4423 pathways or processes.

By extracting functional information from multiple sources, we were able to verify and rank the importance of NOTCH signaling, WNT, inflammatory pathways, nuclear receptor signaling, nuclear degranulation, and cancer pathways to BE and EAC. Out of the 33 genes, 28 were involved in the cancer pathways and processes. The genes that were particularly important in these pathways were *WDR63, GNA12, KAT2B, RBMS3, VPS13B, TLE1, MON2,* and *CTAGE5.* In addition, miR-4423 seemed to have a key role among the identified pathways*.*

**Fig. 5:**
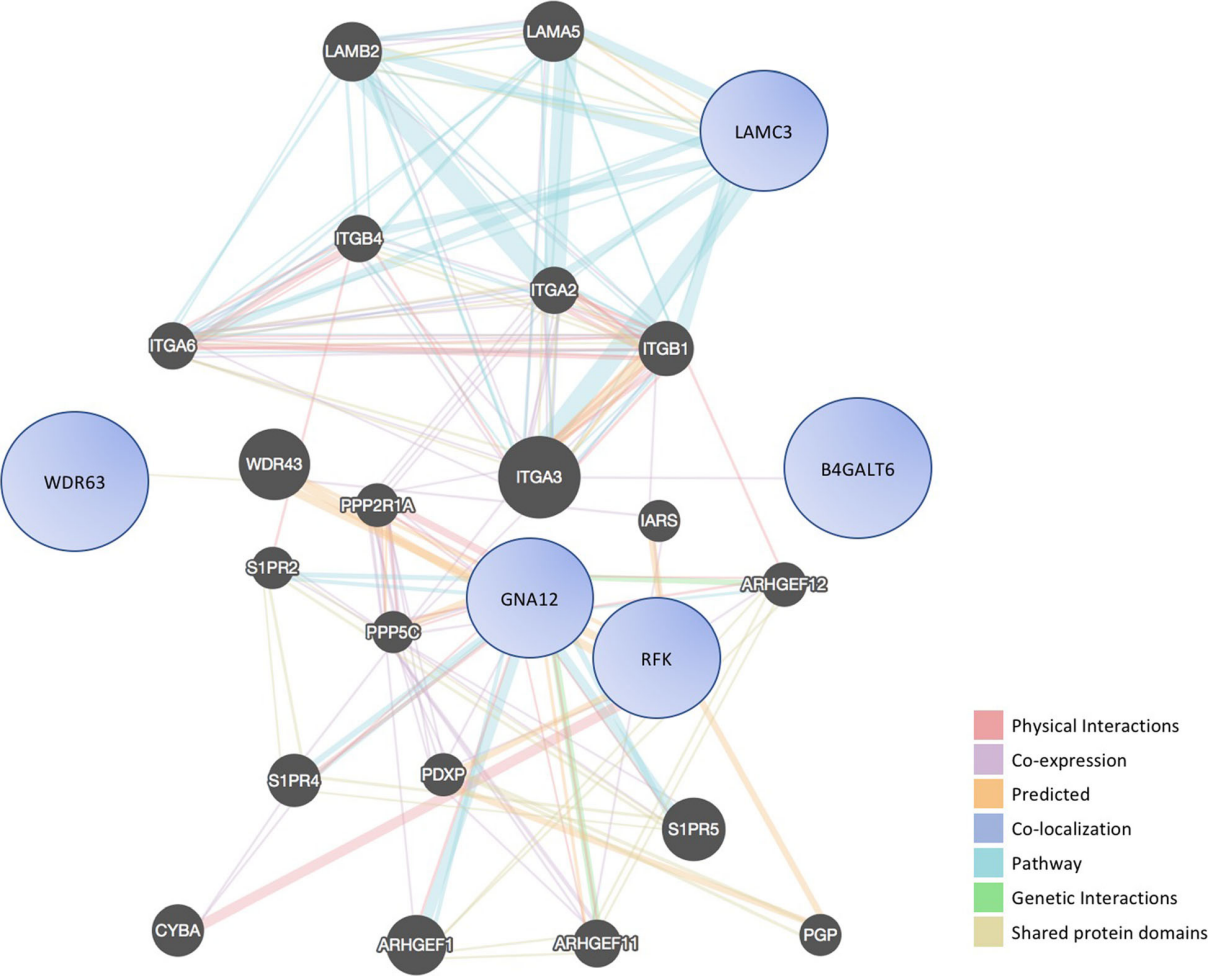
Prioritized networks to predict gene function in BE/EAC. Network interaction analysis and prioritization of genes (i.e., *WDR63, GNA12, RFK, B4GALT6, and LAMC3*) identified that these genes link with each other and are important in BE/EAC cellular processes

We then performed network analysis to identify interactions amongst the 33 genes relevant to BE/EAC, and found that 5 co-expressed genes out of 33 genes (*WDR63, GNA12, RFK, B4GALT6,* and *LAMC3*) were indeed part of the network (Fig. 5). *LAMC3* was involved in extracellular matrix (ECM) receptor interaction and regulation of focal adhesion, which plays an important role in the maintenance of tissue structure and tissue morphogenesis. The interactions between cells and the ECM can regulate cellular activities, such as migration, proliferation, and apoptosis. *GNA12* (G protein subunit alpha 12) was found in the WNT signaling pathway. *GNA12* can be upregulated by GPCR and then trigger RhoGEF, Rho, ROCK, and subsequently affect tissue invasion and metastasis. The second pathway was a metabolic pathway, where *B4GALT6* and *RFK* were involved in glycan biosynthesis and metabolism.

## Discussion

Genetic heterogeneity and the complex BE and EAC clinical outcomes have presented challenges in diagnosis and management of the BE/EAC. Whereas the importance of SNPs and their associations with disease risk are well established, clearly SNPs alone cannot completely unravel the complex link between the genome and the disease. More rigorous and inclusive genomic approaches are warranted to identify global contribution of the diverse genomic alterations in the development of BE/EAC. In this study, we use SNP data to screen for TOHs. Then, we integrated the exome sequence data within the TOH regions to identify IRs or direct repeats and insertion/deletions to prioritize genes and pathways that are important in BE/EAC. Our integrated analysis across platforms revealed genes that play a role in key significant pathways important to BE and EAC development and progression. These pathways were NOTCH, WNT, inflammatory pathways, nuclear receptor signaling, nuclear degranulation, and cancer pathways [21].

We observed that several genes from our list were associated with the development of BE or EAC and replicated previous studies. For example, the MetaCore pathway analysis linked *GNA12* to inflammatory roles in BE/EAC. *GNA12* has previously been shown to be upregulated in esophageal squamous cell carcinoma cells [37], which induces the carcinogenic effects of *GNA12*. Furthermore, GNA12 promotes tumor-cell invasion and metastasis by activating the RhoA/ROCK signaling pathway and upregulating proinflammatory cytokine production [38–41]. Interestingly, *RBMS3*, one of our candidate genes, has previously been shown to have a tumor-suppression function, through c-Myc downregulation, and contributed to poor prognosis in esophageal squamous cell carcinoma [42].

Emerging data now provide insights into the link among methylation of different repeat families, maintenance of chromosome structural integrity, and fidelity of normal transcriptional regulation [27]. Interestingly with the integrated data from IRs, insertion/deletions, and significant cTOHs, we were able to identify key genes that may have a role in BE/EAC. The frequencies of IRs, insertions, and deletions in the case-only exome data implies that the variants maybe important in BE and EAC. More specifically, *WDR63,* miR-4423, *VPS13B, MON2*, and *CTAGE5* consistently showed these overlapping variants. Previous studies have shown that hypomethylation is more prevalent in the repeat regions and has diverse ways of contributing to cancer behavior. For example, hypomethylation of repeated DNA sequences [43–46] is largely responsible for the global DNA hypomethylation that is frequently observed in cancers [47–49]. Tandem centromeric satellite, centromere-adjacent satellite 2, the interspersed Alu, and long interspersed elements (LINE)-1 repeats are the most frequently studied DNA cancer hypomethylated repeats [44–52]. Further study is warranted to assess the nature of methylation patterns in the five key genes we identified and their correlation with the progression of BE to EAC or with the severity of the diseases. BE/EAC-associated aberrations in the miRNA and/or epigenetic patterns can explain the development and clinical stages of the diseases. Along the same lines, miR-4423 has been shown to regulate *WDR63* and has previously been linked to airway epithelial cell differentiation and lung cancer [36]*.*

Our pathway analysis showed similar results compared to previous multi-region whole-exome sequencing studies [53–55]. Chen et al. [53] reported similar pathways, such as the NOTCH signaling pathway and WNT pathway, when comparing a tumorous dysplasia cohort and a non-tumorous dysplasia cohort in mutational landscapes. The NOTCH signaling pathway has been associated with *CDX2* gene expression in the development of BE [56]. Our findings from the REACTOME database also indicate the importance of NOTCH signaling, based on our prioritized list of key genes. A previous publication showed that increased *CDX2* expression [57] is driven by inhibiting NOTCH signaling during BE development [58]. Our MetaCore analysis also showed that *HNF4G* and *TLEI* are two genes that have a role in the NOTCH and WNT pathways and signal transduction. More importantly, *TLEI* and *WDR63* have similar highly conserved C-terminal WD-repeat domains; hence, they will display similar functions. In addition to the independent role of pathways, crosstalk between WNT and NOTCH signaling plays an important role in cancer prognosis. The binding of secreted WNT ligands to the cysteine-rich domain of Frizzled (Fzd) family receptors stimulates the WNT signaling pathway [59], where we found the *GNA12* gene (Fig. 4a).

For other carcinogenesis processes, the interplay of *WDR63* and miR-4423 was reported to be associated with lung cancer [36, 60]. The mature forms of miR-4423 can co-express with *WDR63* in mucociliary epithelium. *WDR63* is downregulated in lung cancers, probably through DNA methylation. MiR-4423 regulates airway epithelium differentiation by repressing the Delta/Notch pathway [36]. Both miR-4423 and *WDR63* can be affected by DNA damage or rearrangement (e.g., due to IRs) and stress-induced transcription factors. Our study is the first to report the possible carcinogenesis function of *WDR63* and miR-4423 among BE/AC patients. Since lung cancer and esophageal cancer share similar risk factors, such as alcohol and tobacco use, and have similar histological subtypes, some genes may play similar roles in different types of cancer development.

## Conclusions

This study highlights the importance of integrating TOH data with IRs to identify DNA rearrangements that can inform BE/EAC development. BE/EAC-specific microRNA expression, measured in readily collected samples, can be used for early BE/EAC detection. This data can be potentially integrated with other ‘omics’ data for a comprehensive understanding of complex susceptibility of BE/EAC.

## Methods

### Selection of BE/ EAC patients

This study was approved by the respective Institutional Review Boards for Research at each participating location where the research was performed. The study involved recruitment of all consenting adults with histological-proven BE and/or EAC as well as families with two or more cases with BE and/or EAC from both academic and community hospitals and clinics. Only white patients of Northern or Western European descent were selected and sex-matched between cases and controls.

#### Genotyping and QC

Germline genomic DNA samples obtained from white blood cells were genotyped using Human610-Quad BeadChips, after which the resulting genotypes were subjected to routine quality control steps: determination of missing genotype rate, testing for non-random genotyping failure, Hardy-Weinberg equilibrium, genotype call rates, MAF of 3–5%, and finally checking for contamination from pipetting errors. Samples were screened and selected only if they had a minimum 95% successful genotype call rate. SNPs with departures from Hardy-Weinberg equilibrium (HWE test, *P* = 0.0000001), and missingness per SNP greater than 5% were excluded from further analyses. As a result, 176 cases/192 controls (231 males/137 females) were kept. We used genotypes from Chr 1~22 only and in total, 570,044 SNPs genotypes were used.

#### Assessment of population stratification

Failure to account for population substructure may lead to both false positive and false negative SNP-disease associations [61]. BE/EAC has been reported to be highly prevalent in populations of European ancestry, but nonetheless, population stratification was analyzed, as previously described [5], by using the principal components analysis (PCA) module contained in EigenStrat [62, 63], and by using PLINK software [64]. Since the population was matched by race, we did not detect population substructure.

### Quantifying tracts of homozygosity and comparing frequencies in cancer cases and controls

#### Identifying TOH and common TOH (cTOH) regions

We used the method described in Orloff et al. [35]. The data from all research participants were examined to determine whether a minimum number of individuals shared a TOH call at a given position (Fig. 5). To identify statistical differences between TOHs within a case-control design, we only retained those TOHs in which 10 or more subjects shared 100 identical homozygous calls, which we operationally define as a common TOH (cTOH). A total of 644 cTOHs were identified across the genome, ranging in size from 100 to 4827 SNPs in length (mean = 196, SD = 221, median = 147, first quartile is 119, and third quartile is 211), and from 136 kb to 15,410 kb (mean = 1160 kb, SD = 1445 kb, median = 793 kb, first quartile is 521 kb, and third quartile is 1194 kb) (16) to identify TOHs.

#### Detection of cTOHs that are associated with BE/EAC

We then pursued testing for association between cTOH and BE/EAC. By considering each cTOH as a genomic variant, a genome-wide case-control analysis was conducted for each cTOH, where a cTOH was viewed as a binary variable based on the presence or absence of a cTOH. A logistic model was fitted for each cTOH by considering disease status as the outcome and the cTOH as the predictor, and we adjusted for gender and population stratification factors. *P*-values were obtained by Wald tests and ORs (95% CI) and were calculated through coefficient estimates of the fitted logistic model (Table 1).

### Analysis of BE/EAC exome and integration with cTOH, insertions, deletions, and nucleotide repeats

Whole-exome libraries from eight independent BE/EAC patients were prepared and sequenced. We followed the exome sequence pipeline from the Broad’s Genome Analysis Tool Kit (GATK Version 3, best practices work flow) [65] to process the sequence data. Raw-exome sequence reads were mapped onto the human reference genome sequence build 36/hg18, downloaded from the University of California, Santa Cruz (UCSC) genome browser with the Burrows-Wheeler aligner [66] (BWA version v.0.6.1; http://bio-bwa.sourceforge.net).

Since the TOH regions likely harbor other types of genomic variants, we sought to identify insertions and deletions using the exome sequence data. Insertion or deletion (indel) realignment, base- and quality-score recalibrations from the resultant binary alignment map (BAM) files were performed with GATK, Sequence Alignment/Map [66] (SAMtools), and Picard. Variant discovery and indel calling were performed with the GATK Haplotype Caller. The high-quality sequences were assembled with the de novo assembler SPAdes, version 3.12.0, and compared with MEGAHIT.

For insertions or deletions to be significant, they had to appear in at least three out of the eight individuals who were sequenced, and they had to overlap with regions carrying inverted or simple repeats. Nucleotide repeat elements are abundant in the human genome and may have significant roles in disease development [47–49]. Therefore, the reference genome was checked for the presence of simple repeats, using RepeatMasker 4.0.7, and IRs, using Inverted Repeats Finder (IRF) version 3.05 [67], to locate and/or predict locations of IRs in the exomic and/or flanking regions of genes located in the TOH regions. The minimap2 was then used to map assembled contigs from eight BE/EAC patient samples that served as a validation series on the reference genome and within cTOH regions.

**Fig. 6:**
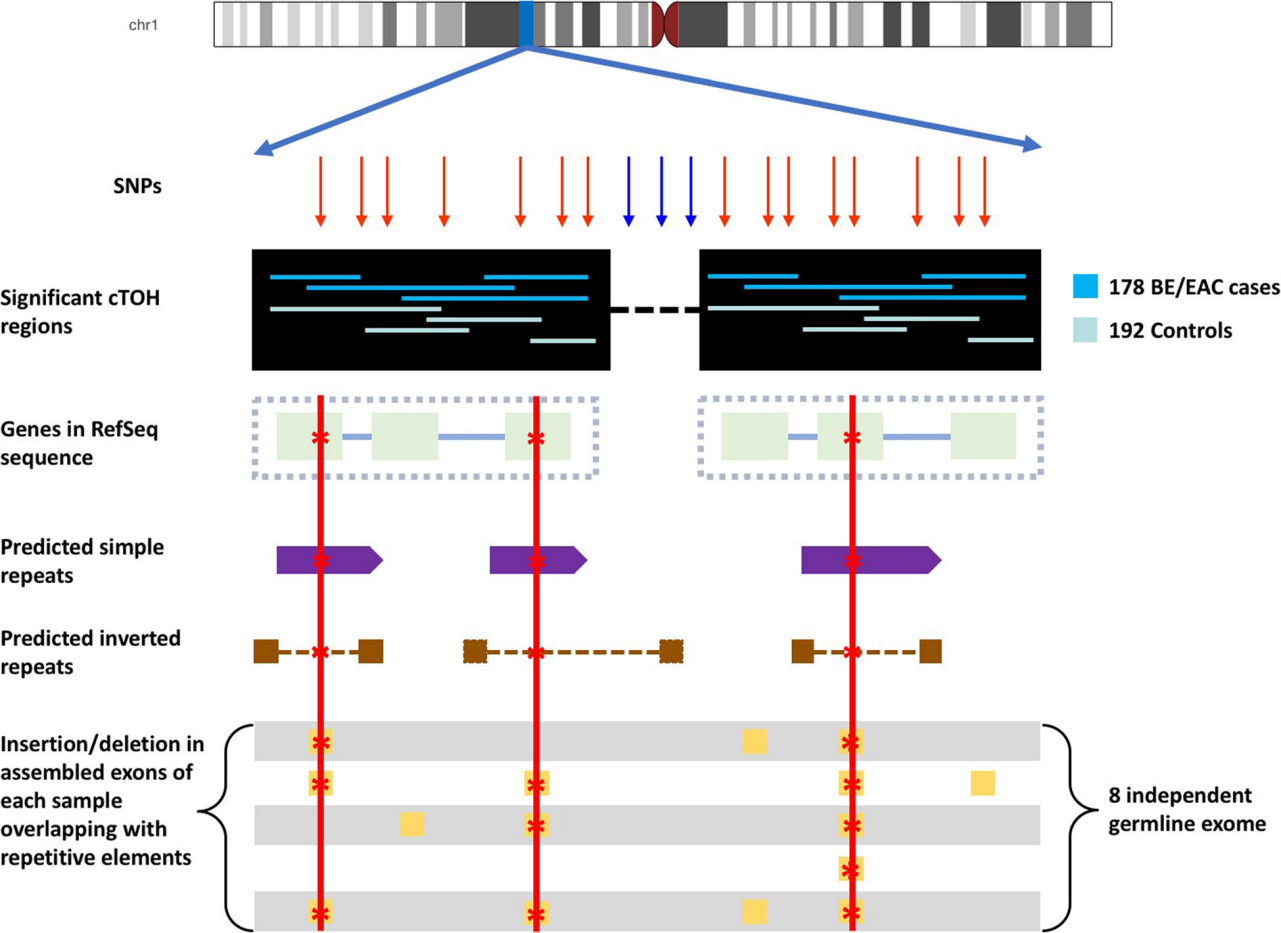
Data analysis workflow from prediction of cTOH regions to validating using exome sequencing data. Overall analysis pipeline of exome sequencing, identification of repeat elements, insertions, deletions, and integrating with TOH data

Bedtools 2.27.0 was used to extract the overlapping regions from all data: reference genes, cTOH regions, nucleotide repeat elements, and contigs from all samples. In-house python scripts were written to automate the analysis pipeline from assembling exome data to mapping repetitive elements to identifying cTOH blocks and insertions/deletions for all eight germline samples (Fig. 6). We inspected all resultant variants through the Integrative Genomics Viewer [68] (IGV; https://software.broadinstitute.org/software/igv/). The genes associated with BE/EAC and containing insertion/deletion within contigs across all samples were collected in a tab-separated value (TSV) file and visualized using R packages: ggplot2 and ggbio.

### Pathway and network analyses to predict functional roles in BE/EAC

Since the key genes identified within cTOH regions that overlapped with the insertions/deletions and IRs may have possible roles in either the development or progression of BE to EAC, we used MetaCore bioinformatics software and curated Comparative Toxicogenomics Database to analyze biological pathways as well as disease and gene networks that are associated with BE/EAC. MetaCore contains an integrated pathway and network analysis for multi-omics types of data and also has a comprehensive systems biology analysis suite that helps identify high-quality experimental molecular interactions and pathways, gene disease associations, as well as chemical metabolism and toxicity information.

Network analysis was done using an open source GeneMANIA package, which builds and uses weighted gene interaction networks from various sources of data [69]. It uses a fast heuristic algorithm, derived from ridge regression, to integrate multiple functional association networks and predict gene function from a single process-specific network using label propagation. Genes that were significant from our TOH and exome analyses were analyzed to predict possible roles in BE and/or EAC.

## Additional file

Additional file 1: Locations for genes within cTOH regions that also harbor indels. (XLSX 14 kb)

## Abbreviations

BE: Barrett’s esophagus
cTOH: Common Tracts of homozygosity
EAC: Esophageal cancer
GATK: Genome Analysis Tool Kit
TOH: Tracts of homozygosity
